# Changing ideas about others’ intentions: updating prior expectations tunes activity in the human motor system

**DOI:** 10.1038/srep26995

**Published:** 2016-05-31

**Authors:** Pierre O. Jacquet, Alice C. Roy, Valérian Chambon, Anna M. Borghi, Roméo Salemme, Alessandro Farnè, Karen T. Reilly

**Affiliations:** 1 Evolution and Social Cognition Group, Laboratoire de Neurosciences Cognitives (LNC), Département d’Etudes Cognitives, INSERM U960, Ecole Normale Supérieure, PSL Research University, F-75005 Paris, France; 2 Evolution and Social Cognition Group, Institut Jean Nicod, Département d’Etudes Cognitives, CNRS UMR8129, Ecole Normale Supérieure, PSL Research University, F-75005 Paris, France; 3 Dynamique Du Langage, CNRS UMR 5596, University Lumières Lyon II, 69363 Lyon, France; 4 Department of Neuroscience, Biotech Campus-University of Geneva, 1211 Geneva, Switzerland; 5 Department of Psychology, Bologna University, 40127 Bologna, Italy; 6 Institute of Sciences and Technologies of Cognition, CNR, 00185 Rome, Italy; 7 ImpAct Team, Lyon Neuroscience Research Center CRNL, INSERM U1028, CNRS UMR5292, & Hospices Civils de Lyon, Neuro-immersion & Mouvement et Handicap, 69675 Bron, France; 8 University Claude Bernard Lyon I, 69100 Villeurbanne, France

## Abstract

Predicting intentions from observing another agent’s behaviours is often thought to depend on motor resonance – i.e., the motor system’s response to a perceived movement by the activation of its stored motor counterpart, but observers might also rely on prior expectations, especially when actions take place in perceptually uncertain situations. Here we assessed motor resonance during an action prediction task using transcranial magnetic stimulation to probe corticospinal excitability (CSE) and report that experimentally-induced updates in observers’ prior expectations modulate CSE when predictions are made under situations of perceptual uncertainty. We show that prior expectations are updated on the basis of both biomechanical and probabilistic prior information and that the magnitude of the CSE modulation observed across participants is explained by the magnitude of change in their prior expectations. These findings provide the first evidence that when observers predict others’ intentions, motor resonance mechanisms adapt to changes in their prior expectations. We propose that this adaptive adjustment might reflect a regulatory control mechanism that shares some similarities with that observed during action selection. Such a mechanism could help arbitrate the competition between biomechanical and probabilistic prior information when appropriate for prediction.

Predicting and understanding other people’s behaviours is often thought to depend on processes of motor simulation or resonance^1^,^2^,^3^,^4^,^5^,^6^,^7^,^8^,^9^,^10^,^11^. Motor resonance refers to the transformation of observed movement kinematics into self-centered motor counterparts^12^ within the human mirror neuron system or action-observation network, i.e., a parieto-frontal network comprising visuo-motor neurons discharging both during action execution and observation. Some researchers, however, question the idea that resonating visuo-motor parameters of an observed actions is the only source of information used to attribute the agent’s underlying intention^13^,^14^,^15^,^16^,^17^,^18^. One reason for this is that motor resonance alone cannot satisfactorily deal with the uncertainty often met by an observer when interpreting other people’s behaviours^14^,^19^,^20^. Indeed, to successfully predict another agent’s behaviour the observer needs to deal with variations in observational contexts by exploiting, adjusting, or inhibiting certain information. Solving this uncertainty problem can be made easier by our ability to form prior expectations about other agents’ behaviours and intentions, i.e., expectations about how an agent is likely to behave in a given situation^14^,^21^,^22^.

Prior expectations can be modeled as the product of various sources of prior information. One source is the probabilistic information an observer can extract from the past distribution of an agent’s behaviour^23^. Another source is the amount of biomechanical effort (or cost) used by an agent in her/his goal-directed actions. Mounting evidence shows that humans exhibit strong expectations about the biomechanical ‘optimality’ of goal-directed motor behaviours both in an execution and an observation context^24^,^25^,^26^,^27^,^28^. Variations in observed or planned biomechanical effort have also been shown to be selectively encoded within the motor system of both non-human^29^,^30^ and human primates^31^. When human agents intend to hit a nail using a hammer for example, they tend to choose the action means that requires the least joint and muscular effort given the properties of the tool. Reciprocally, human observers expect other agents to exploit these ‘optimal’ action means to achieve their goals. In some situations however, probabilistic and biomechanical prior information may diverge from each other. An observed agent may occasionally find it beneficial to not follow biomechanical optimality rules. For example, an athlete might use an unfamiliar, biomechanically awkward motor strategy at a certain frequency and then suddenly change strategies in an attempt to deceive his opponent. Tracking biomechanical and probabilistic information and solving the potential conflicts between them enables human observers to narrow the number of intentions (or goals) that compete to explain other people’s behaviour. This results in the selection of intentions that the observed agent is more likely to pursue given their prior distributions^21^,^22^,^32^, the biomechanical constraints of the effector^33^, and the physical properties of the objects that might be used to achieve their goal^34^. To date, only a few studies have tested the contribution of biomechanical and probabilistic prior information to the prediction of action intentions^21^,^22^,^34^, and none have investigated their impact on motor resonance mechanisms.

In this study we examined how experimentally biasing prior expectations about another agent’s behaviour affects motor resonance during the prediction of tool-use actions. Healthy adult participants were asked to make predictions about an agent’s final intentions or goals (in the following manuscript we will interchangeably use these two terms to refer to the mentalized and actualized end-state of the action) while watching movies in which the agent grasped an unfamiliar tool with two different grips (power or precision) and then manipulated it to achieve two different goals (open the box or turn on the light). The tool was designed such that grasping it using a power grip with the aim of opening the box required less biomechanical effort than achieving the same goal using the precision grip. Likewise, grasping the tool with a precision grip with the aim of turning on the light required less biomechanical effort than achieving the same goal using the power grip. Thus, among the four possible action combinations, two were biomechanically optimal and two were suboptimal. We also varied the amount of visual information provided by the movies in such a way that in one condition only the type of grip was visible (goal-hidden movies) while in another condition both the grip and the final goal were visible (goal-visible movies). Unbeknownst to participants, during the block of goal-visible movies we experimentally biased their expectations about the goal the agent would achieve using a particular grip. To do so, the probability of viewing the agent using each grip to achieve the goal in a biomechanically optimal or suboptimal manner was varied, while the probability of viewing a given goal did not vary. Participants were randomly assigned to one of three groups: NO BIAS (goals achieved using optimal and suboptimal kinematics were shown equally during the goal-visible movies); CONVERGENT BIAS (goals achieved using optimal kinematics were shown more frequently); DIVERGENT BIAS (goals achieved using suboptimal kinematics were shown more frequently). To investigate whether and how, this manipulation affected participants’ predictions in condition of visual uncertainty we showed them two blocks of goal-hidden movies, before and after being exposed to one of the three probability biases. In both goal-hidden and goal-visible movie blocks, prior expectations and their trial-by-trial updates were calculated using a simple Bayesian learning scheme. From this we measured an averaged Response Bias (RB) which indicated preference for predicting intentions congruent or incongruent with biomechanical optimality. During the two goal-hidden movie blocks (one before and one after exposure to biased probabilities) motor resonance^35^,^36^ was assessed by probing corticospinal excitability (CSE) using single-pulse transcranial magnetic stimulation (spTMS). We expected that all participants would initially show a RB towards biomechanical optimality. We further expected this RB to be updated as a function of the probability distribution of the kinematic strategies to which they were exposed in the goal-visible movie block. If, as we hypothesize, the motor resonance system is sensitive to prior expectations and their updating – then CSE should be modulated by the type of probabilistic bias and not the grip (which was similar across all groups), and this effect should be greater for participants who update their prior expectations. Conversely, if motor resonance is specific for movement kinematics, then CSE should be unaffected by exposure to biased probabilities.

## Results

Statistical analyses were performed using Statistica 8 (www.statsoft.com) and Matlab v.R2012b (The MathWorks). All post-hoc pairwise comparisons were carried out using Newman-Keuls test and one-tailed t-tests. A significance threshold of P < 0.05 was set for all statistical tests.

### Prior expectations (Response Bias [RB])

#### Bias block (goal-visible movies)

**Figure 1:**
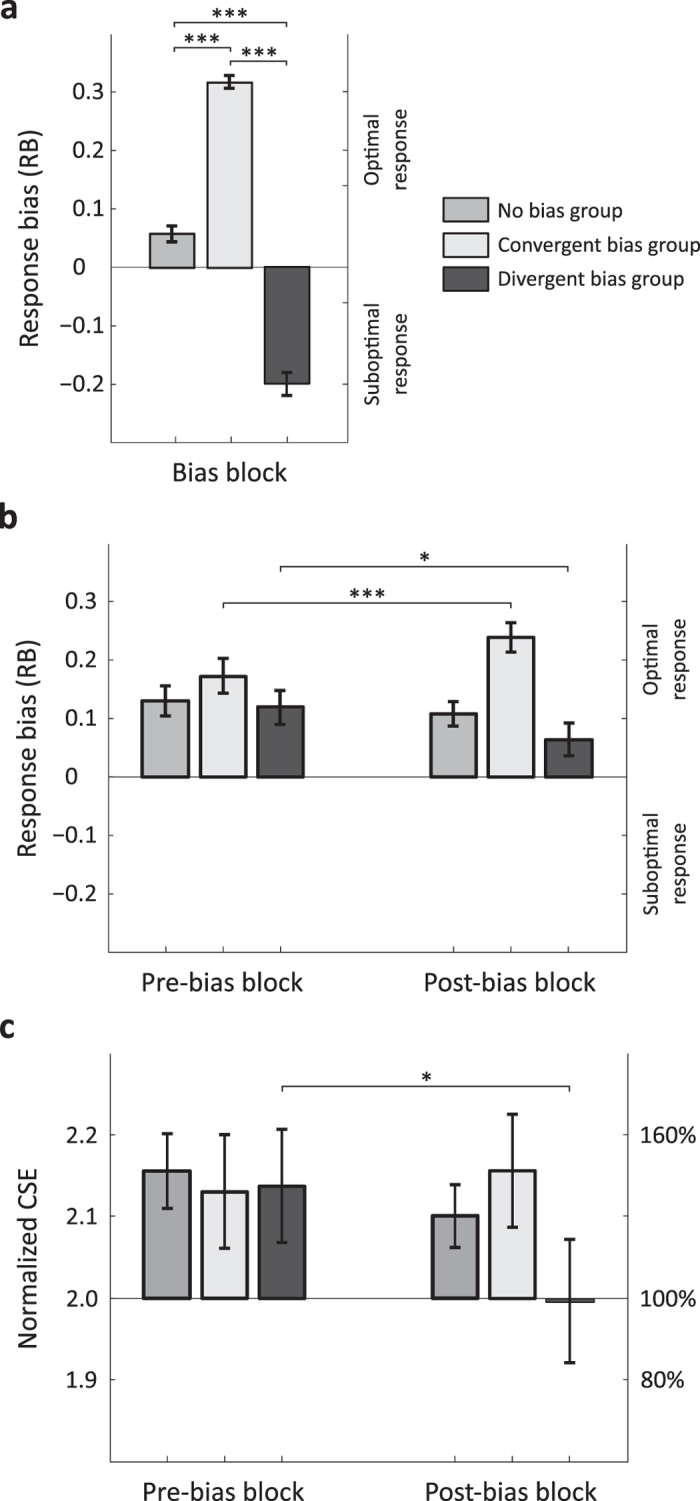
Prior expectations and CSE. (**a)** Prior expectations (±SEM) measured as the mean Response Bias (RB) for the bias block (goal-visible movies) for each of the three groups (no bias, convergent bias, divergent bias). (**b)** Prior expectations (±SEM) measured as the mean Response Bias (RB) for the pre- and post-bias blocks (goal-hidden movies) for each of the three groups (no bias, convergent bias, divergent bias). Deviations from 0 indicate the presence of a response bias for intentions congruent with optimal (greater than 0) or suboptimal kinematics (less than 0). (**c)** Mean normalized CSE (±SEM) in the pre-bias and post-bias blocks for each of the three groups. The normalized CSE (on the left vertical axis) represents the log-transformed percentage of mean MEP amplitude recorded during the pre-bias and post-bias blocks relative to the mean MEP amplitude recorded during the baseline blocks (corresponding untransformed percentages are shown on the right vertical axis). Deviations from 2 (100%) indicate an increase (>2) or decrease (<2) in CSE during action prediction. Asterisks indicate significant comparisons (*p < 0.05; **p < 0.01; ***p < 0.001).

To investigate whether participants updated their prior expectations in line with the probability distributions to which they were exposed, we analysed the mean RB values collected during the bias block using a 2 × 3 repeated-measures ANOVA with ‘grip’ (power, precision) as a within-subject factor and ‘group’ (no bias, convergent bias, divergent bias) as a between-subject factor. The middle column of Table 1 shows the response bias for the goal-visible block separately for each grip and for each of the three groups and Fig. 1a shows these same data averaged across the two grips. The ‘grip’ × ‘group’ ANOVA performed on the mean RB values revealed a main effect of group (F_2,41_ = 283.21, p < 0.001): compared with the no bias group (RB = 0.06 ± 0.05), the convergent bias group had a higher RB (0.32 ± 0.038, p < 0.001) while the divergent bias group had a lower RB (−0.20 ± 0.08, p < 0.001). Importantly, all three RB’s differed from zero (one tailed t-tests compared to 0; convergent: t = 32.44, p < 0.001, divergent: t = −9.2, p < 0.001, no bias: t = 4.37, p < 0.001), indicating that participants encoded the probabilities present in the goal-visible block and adapted their predictions accordingly. This analysis shows that 1) participants in the no bias group showed a slight preference for predicting intentions congruent with optimal kinematics (preference for the ‘open the box’ intention when a power grip was presented, and for the ‘turn-on the light’ intention when the precision grip was presented), 2) this preference for predicting optimal kinematics was strengthened for participants exposed to the convergent bias, and 3) participants exposed to the divergent bias preferred predictions congruent with a suboptimal kinematic strategy (‘open the box’ intention when a precision grip was presented, and ‘turn-on the light’ intention when the power grip was presented). Note that between-group differences in RB went hand-in-hand with between-group reaction time differences (see Supplementary Material for a detailed analysis RTs). Specifically, participants in the no bias and convergent bias groups had faster RTs for their preferred responses (optimal). Crucially, the absolute values of the RB for the convergent (0.32 ± 0.038) and divergent (−0.20 ± 0.08) groups were significantly different (one-tailed t-test, t > 5.95, p < 0.0001). The greater RB for the convergent group was notably due to the fact that participants made more recognition errors for intentions achieved with suboptimal kinematics (see Supplementary Material and Supplementary Fig. S1 for a detailed analysis of errors). This indicates that participants exposed to the convergent bias had difficulties inhibiting the prepotent ‘optimal’ response, and so in spite of the fact that i) the probability of observing intentions achieved with ‘optimal’ actions in the convergent bias was equal to the probability of observing intentions achieved with ‘suboptimal’ actions in the divergent bias (i.e., 80% vs 20%), ii) there was enough visual information to unambiguously identify between the two alternatives.

#### Pre-bias and post-bias blocks (goal-hidden movies)

In order to assess the effect of the induced probabilistic bias on participants’ prior expectations in conditions of perceptual uncertainty we analysed the mean RB values from the pre-bias and post-bias blocks using a 2 × 2 × 3 repeated-measures ANOVA with ‘grip’ (power, precision) and ‘block (pre-bias, post-bias) as within-subject factors and ‘group’ (no bias, convergent bias, divergent bias) as a between-subject factor. The first and last columns of Table 1 show the response bias for the pre- and post-bias blocks separately for both grips and for each of the three groups. Figure 1b shows these same data averaged across the two grips. The first thing to notice is that in the pre-bias block RB was similar for all three groups (all ps > 0.21) and was significantly greater than zero (one tailed t-tests compared to 0; all ts > 4.33, all ps < 0.001). Thus, before being exposed to biased or unbiased probabilities participants made predictions that were directed *by default* towards intentions consistent with optimal kinematic strategies (‘open the box’ when a power grip was presented, and ‘turn-on the light’ intention when a precision grip was presented). This *by-default* prediction mode is also apparent in the error analysis (see Supplementary Material and Supplementary Fig. S1). This was not the case in the post-bias block. Indeed, the ‘grip’ × ‘block × ‘group’ ANOVA revealed a significant main effect of ‘group’ (F_2,41_ = 7.60, p < 0.01) and a significant interaction between ‘group’ and ‘block’ (F_2,41_ = 10.43, p < 0.001) (see Fig. 1b). While no difference in RB was observed between the three groups in the pre-bias block, in the post-bias block there was a significant difference between the convergent group and the two other groups (all ps < 0.01). The no bias group’s positive RB (consistent with predicting intentions congruent with optimal kinematic strategies) was unchanged by exposure to the goal-visible movies (0.13 ± 0.07 vs 0.11 ± 0.08, p > 0.53), but for both the convergent and divergent bias groups RB changed significantly. For the convergent bias group, RB increased significantly in the post-bias block (0.17 ± 0.10 vs 0.24 ± 0.08; p < 0.01), indicating that participants shifted their bias even further towards intentions congruent with optimal kinematic strategies. In other words, when they saw the power grip they more often predicted the ‘open the box’ intention than the ‘turn on the light’ intention, and vice versa when they saw the precision grip. The reverse was true for the divergent bias group, who shifted their RB towards intentions congruent with suboptimal kinematic strategies, as shown by the fact that RB decreased significantly in the post-bias block (0.12 ± 0.11 vs 0.06 ± 0.09, p < 0.05). In the goal-visible block, participants in this group were clearly biased towards intentions congruent with suboptimal kinematic strategies. Interestingly, while RB in the post-bias block was significantly smaller than in the pre-bias block, RB differed from zero only for the pre-bias block, indicating that after exposure to the divergent bias they had no preference for either optimal or suboptimal responses (see also the analysis of error rates in the Supplementary Material).

### Corticospinal excitability (CSE)

#### CSE modulation by exposure to biased probabilities (normalized MEPs)

To determine whether exposure to the different probability distributions affected CSE during predictions made under conditions of visual uncertainty we analysed the normalized mean motor-evoked potentials (MEP) amplitudes using a 2 × 2 × 2 × 3 repeated-measures ANOVA with ‘grip’ (power, precision), ‘block’ (pre-bias, post-bias), and ‘response type’ (optimal, suboptimal) as within-subject factors and ‘group’ (no bias, convergent bias, divergent bias) as a between-subject factor. Table 2 shows the log-transformed normalized MEP amplitudes for the pre- and post-bias blocks separately for each observed grip, each response type and for each of the three groups and Fig. 1c shows these same data averaged across the two grips (note that in the pre-bias block CSE was similar across the three groups - all ps > 0.64). The ANOVA revealed a main effect of block (F_1,41_ = 5.18, p < 0.05), with greater CSE in the pre-bias than the post-bias block (2.14 ± 0.24 vs. 2.09 ± 25). Neither response type nor grip modulated CSE or interacted with any other factors. Similar to the RB results, however, there was a significant interaction between the factors ‘block’ and ‘group’ (F_2,41_ = 3.55, p < 0.05), as CSE decreased significantly between the pre- and post-bias blocks in the divergent bias group only (2.14 ± 0.26 vs. 1.99 ± 0.29, p < 0.05). To investigate the possibility that this interaction effect was a false positive (i.e., uncontrolled alpha inflation in the ANOVA’s results caused by the numerous possible interactions between our 4 factors) we performed a Monte-Carlo permutation analysis on the CSE data^37^. We first created 10000 fully permutated data sets. On each data set we then ran the four-way ANOVA and compared the ‘block’ × ‘group’ interaction F value with that obtained with the real, non-permutated CSE data. This revealed that the probability of obtaining an F value greater than the one obtained with the real, non-permutated CSE data was only 2.8%, suggesting that our interaction effect was not a false positive. Finally, it is important to note that a CSE value of 2 corresponds to the baseline level of CSE and that for both the no bias and the convergent bias groups, but not for the divergent bias group the mean CSE level in the post-bias block was significantly greater than 2 (one tailed t-tests compared to 2; no bias: t = 2.56, p = 0.02; convergent: t = 2.21, p = 0.04; divergent: t = −0.02, p = 0.98). This suggests that motor resonance processes were disrupted in the condition where probabilistic information conflicted with biomechanical priors.

#### CSE modulation by changes in prior expectations (normalized MEPs)

**Figure 2:**
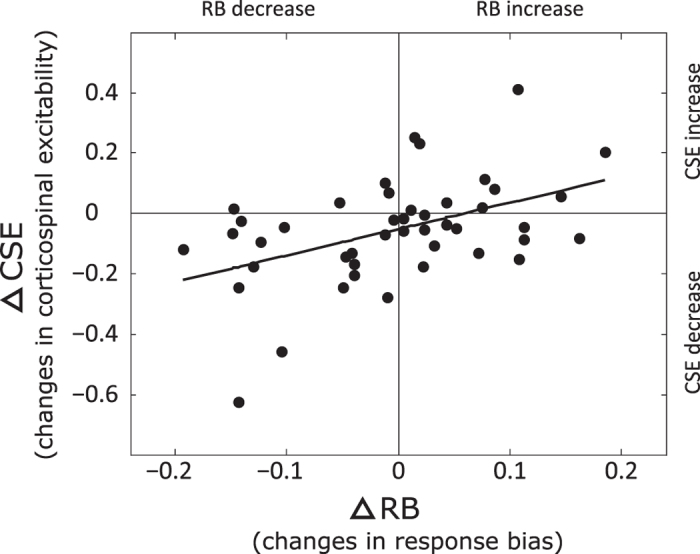
Changes in prior expectations predict changes in CSE. The positive linear relationship between ΔCSE and ΔRB across participant in all three groups (β = 0.47, p = 0.001). A deviation above or below 0 on the Y axis indicates an increase or a decrease in CSE in the post-bias block compared with the pre-bias block; a deviation above or below 0 on the X axis indicates an increase or decrease in RB in the post-bias block compared with the pre-bias block.

In a final analysis we aimed to test whether changes in response bias (ΔRB) predicted changes in CSE (ΔCSE), and whether the direction and strength of this relationship varied according to the type of probabilistic bias to which participants were exposed. To do this, for each participant we first calculated the change in CSE between the pre- and post-bias blocks (averaged across the two grips) and the change in RB between these two blocks (ΔRB) (averaged across the two grips). A positive ΔRB indicates that expectations of observing the agent achieving his intentions using optimal kinematics increased in the post-bias block, whereas a negative ΔRB indicates that these expectations decreased in the post-bias block in favour of the opposite strategy. We then conducted a general linear model with the ‘ΔCSE’ entered as the *dependent* variable, the ‘ΔRB’ as the *continuous* predictor, and the ‘group’ (no bias, convergent bias, divergent bias) as a three-level *categorical* predictor. We used a full factorial design to assess the respective contributions of the continuous and categorical predictors as well as their interactions. The general linear model (adjusted R^2^ = 0.24, F = 3.72, p < 0.01) revealed a main effect of the continuous predictor ‘ΔRB’ (changes in prior expectations) over the dependent variable ‘ΔCSE’ (changes in normalized CSE) (F_1,38_ = 8.29, p < 0.01). This effect was characterized by a positive linear relationship between the two variables, such that the more prior expectations changes, the more corticospinal excitability changed (β = 0.47, p = 0.001) (see Fig. 2). Crucially, the predictive value of ΔRB over ΔCSE was not further modulated by ‘group’ (F_2,38_ = 2.42, p > 0.10). This is of primary importance because it indicates the presence of a general regulatory mechanism of CSE which is driven by incoming information about the observed agent’s likely kinematic strategy.

## Discussion

The aim of this study was to test whether biasing prior expectations about other people’s behaviour modulates activity in the observers’ motor system in accordance with their overt predictions. When exposed to movies in which the agent’s goal was not visible participants initially predicted intentions congruent with biomechanical optimality, i.e. they expected the power grip to predict ‘open the box’ and the precision grip to predict ‘turn on the light’. This default response bias (RB) pattern was significantly altered during the experiment as participants adaptively updated their prior expectations in accordance with the type of probabilistic bias to which they were exposed in the goal-visible movie block (see Fig. 1a,b). This change was paralleled by a change in mean corticospinal excitability (CSE), with a CSE decrease in the group exposed to the divergent probabilistic bias (i.e., where the agent’s kinematic strategy did not match biomechanical priors) and no change in CSE in the two other groups (see Fig. 1c). Importantly, a significant percentage of the variation in the change in CSE across all participants was accounted for by the change in their response bias. Participants with the greatest shift in prior expectations (increase or decrease in RB) were those who exhibited the greatest change in CSE (increase in CSE in the case of an increase in RB vs. decrease in CSE in the case of a decrease in RB). Overall, these results show that the motor system tunes its activity during prediction to reflect the type of prior information that is preferentially exploited for prediction (Fig. 1c). In addition, our data demonstrate that the motor system can adjust its excitability according to changes in prior expectations about other people’s behaviours (Fig. 2). We propose that this adjustment might reflect a regulatory control mechanism that shares some similarities with that observed during action selection^38^. In the present case, however, this mechanism does not operate at the level of action representations. Instead, the CSE modulation appears to reflect the observer’s capacity to disengage from biomechanical priors when they conflict with probabilistic information. That is, when biomechanical priors are irrelevant for predictions.

Our action stimuli evoked a motor facilitation effect (see results section and Supplementary Material), as average CSE was greater during the observation of action stimuli (pre-bias block where movies showed the agent using either a power or precision grasp) than during non-action stimuli (baseline blocks where movies showed a white fixation cross in the middle of a black screen). Despite the fact that our target muscle (FDI) was differentially recruited during execution of the two grips (see material and methods section) the amount of motor facilitation was similar for the two grips (precision and power) (see Supplementary Material). This finding is consistent with the literature, as even though pure motor resonance is defined as changes in CSE that reflect both action specificity and motor facilitation^35^,^36^,^39^,^40^, most studies report only one of these two changes^41^,^42^,^43^,^44^,^45^,^46^,^47^.

While the absence of action specificity in our data does not question the presence of motor facilitation, it does raise the question of the nature of the motor facilitation effect we observed, as it has been shown that motor facilitation can occur as a consequence of non-specific factors such as increased attentional demands or task difficulty instead of pure motor resonance^48^,^49^,^50^. Here, task difficulty and attentional demands decreased across time for participants in the convergent bias group (who simply relied on *by-default* biomechanical priors to make adaptive predictions in the post-bias block) and increased for participants in the no bias and divergent bias groups (who had to inhibit, at least partially, *by-default* priors in order to make adaptive predictions). Thus, if the motor facilitation we observed was a consequence of increasing attentional demands or task difficulty instead of motor resonance then it should have been more pronounced in the post-bias phase for the no bias and divergent bias groups and less pronounced in the convergent bias group, but this was not the case.

The concordance between our CSE and RB data lead us to favour the perspective that when participants have to predict uncertain action goals the motor system’s activity can be tuned to higher-order information instead of on-line kinematics. This perspective is supported by a number of studies on human^3^,^4^,^5^,^7^,^8^,^51^,^52^,^53^,^54^,^55^ and non-human primates^1^,^6^,^9^,^10^,^11^,^56^ which demonstrate that prior knowledge an observer has about the occurrence (or the non-occurrence) of another agent’s behaviours can modulate resonance activity of the motor system. This view is also in line with a number of recent studies suggesting that the contribution of the human mirror neuron system (which subserves motor resonance) to the processing of other people’s action intentions is crucial only under conditions where no general expectations are generated prior to action observation^46^,^57^,^58^,^59^,^60^,^61^,^62^,^63^. In situations where this condition is not met (such as in the present task), activity in the motor system could be influenced by other cortical regions (i.e., midline cortical structures including the posterior cingulate cortex, frontal and parietal regions as well) which play a key role in the generation of prior knowledge about upcoming future events^64^.

Our results are also coherent with data from the domain of action selection, where recent TMS experiments have found evidence for the involvement of inhibitory circuits in M1 for response control during the selection and the preparation of motor actions (for recent reviews, see^65^,^66^). For instance, in a stop signal reaction time task, CSE was reduced when participants successfully delayed or cancelled an impending action following an imperative ‘STOP’ cue^67^. This suggests that such regulatory mechanisms can be driven by the maintenance of internal goals or the integration of acquired rules^68^,^69^ that help individuals make decisions in changing, open-ended environments^70^,^71^. Direct evidence for this comes from recent work showing that during action selection motor cortex activity is sensitive to top-down information pertinent to decisions, such as the participants’ own preferences^72^, the accumulation of sensory evidence^73^, the computation of action value^74^, the prior distribution of action alternatives^75^,^76^,^77^,^78^,^79^ or even their biomechanical cost^28^. Together, these data support the hypothesis that decisions about actions can be made within the motor system via a biased competition between representations of action alternatives^38^.

Our results open the interesting, to date unexplored, possibility that a mechanism that shares some similarities with that observed during action selection is at play during the prediction of other people’s actions. The main – albeit crucial – difference is that such a mechanism does not operate at the level of single action representations. In fact, what our results do show is that i) competition between two types of prior information (i.e., biomechanical vs. probabilistic) can alter expectations about the observed agent’s behavior, and that ii) achieving a trade-off or compromise between the two priors appears to lead to a suppression or a maintenance of motor resonance activity depending on whether these priors compete (divergent bias group) or not (convergent bias group).

A challenge for future studies will be to demonstrate whether the updating of prior expectations can have a modulatory effect on motor resonance activity on a trial-by-trial basis. The inherent variability in MEP amplitudes will make this very challenging, but if it can be shown then one could speculate that changes in prior expectations might trigger a regulatory control mechanism within M1 whereby the weight of action representations consistent with biomechanical prior information would recursively increase or decrease^58^ depending on whether these representations match or conflict with current and past observations. Interestingly, recent work suggests that a similar mechanism is involved in the control of automatic motor imitation, as the anterior cingulate cortex and the medial parts of the frontal cortex modulate inferior frontal gyrus activity^80^,^81^ with direct consequences on the activation threshold of motor representations at play during motor resonance^45^.

Since the automatic activation of motor representations is often not suitable for predicting unexpected behaviours, regulating motor resonance processes through higher-order probabilistic representations of the environment may provide an adaptive mechanism to understand and acquire unexpected and new behaviours^58^,^82^. Remarkably, behaviours that over-ride rules of biomechanical optimization are regularly found in human cultural praxes, as in some forms of sport, dance, or music. In these cases, biomechanical suboptimality is often perceived as the hallmark of excellence, and for that reason is socially rewarded. In order to predict, understand, appreciate or acquire such praxes, relying on prior expectations acquired from past observations and inhibiting motor representations may prove more helpful than merely evaluating the (biomechanical) optimality of the observed action.

## Methods

### Participants

Forty-six healthy volunteers (26 women) aged 19–36 (mean = 24, SD = 4.3) took part in an experiment during which they were asked to predict or recognize the final intentions of a filmed agent manipulating an unfamiliar tool in different ways. All were right-handed according to a standard handedness inventory^83^, naïve to the purpose of the experiment, and reported normal or corrected-to-normal visual acuity. All participants gave written informed consent and received payment for their participation. The experimental protocol was performed with approval of the local Ethical Committee (CPP SUD EST IV) and in accordance with the Declaration of Helsinki (World Medical Association, 2008). None of the participants had any neurological, psychiatric, or other medical problems that are contraindicated for TMS^84^. Participants were randomly assigned to one of three groups (see below) and the groups were matched for age (two-tailed t-test, all ts < 0.30, all ps > 0.20) and resting motor threshold (two-tailed t-test, all ts < 01.33, all ps > 0.11).

### General Procedure

Participants sat in front of a 19-inch computer screen and watched 288 movies ([2 × 96 goal-hidden] + [1 × 96 goal-visible]) and corticospinal excitability (CSE) was measured during the goal-hidden movies using single pulse TMS. The movies lasted 2000 ms (30 frames per second, subtended 35° of visual angle) and showed a male agent using his left hand to act upon an unfamiliar handle located on top of a box. Participants were required to predict (in the case of goal-hidden movies) or recognize (in the case of goal-visible movies) the agent’s intention by saying ‘A’ if they thought the agent was going to open the box or ‘B’ if they thought he was going to turn on the light. They were instructed to respond as soon as they thought they had enough visual information to produce an accurate response and their responses were recorded on-line with a microphone. At the end of each movie their vocal response time (RT) – calculated from the onset of the movie – was displayed on the screen for 500 ms. They were explicitly asked to use this feedback to monitor their performance and avoid responding before the goal-hidden movies froze (800 ms after onset) or before the goal was clear in the goal-visible movies as responding too early would have hindered the integration of the probabilistic bias during the goal-visible movie block. If participants did not respond, ‘NO RESPONSE’ was displayed on the screen for 500 ms and the next trial began 2500 ms later (the average number of missed trials was about 1.9%, that is, 2.2 trials per block).

Prior to the experimental session participants were familiarised with the task by watching six goal-hidden movies and twelve goal-visible movies (containing six optimal and six suboptimal actions). The presentation of the stimuli, recording of vocal responses, and TMS triggering were controlled using *Presentation* software (Neurobehavioral Systems, Inc., USA).

### Biomechanical effort associated with action stimuli

**Figure 3:**
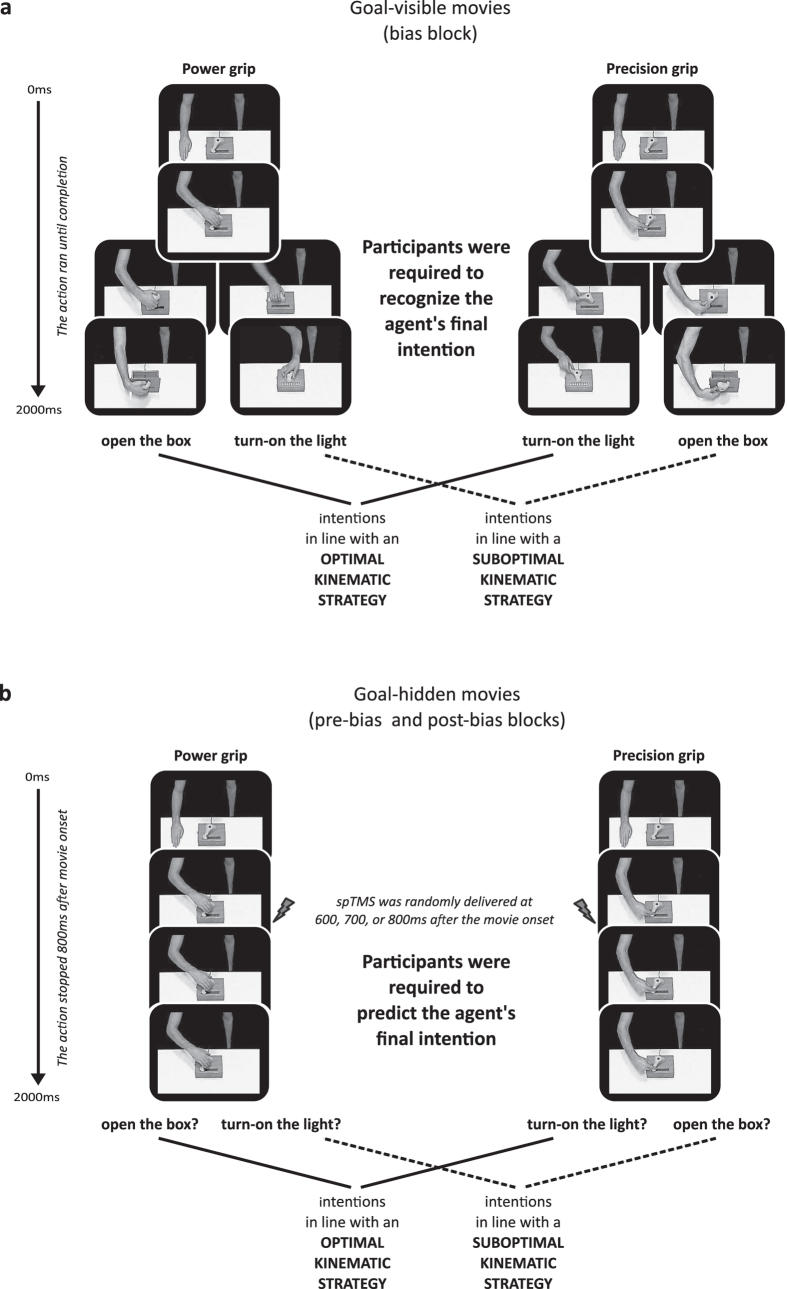
Examples of the four combinations ‘grip × goal that participants saw during the experiment. All combinations began with the agent’s static hand. (**a)** The agent could then manipulate the tool using either a ‘power’ or a ‘precision’ grip with the intention of either opening the box or turning on the light. The tool was designed such that grasping it using a power grip with the aim of opening the box elicited less biomechanical effort than achieving the same goal using the precision grip. Likewise, grasping the tool with a precision grip with the aim of turning the light on elicited less biomechanical effort than achieving the same intention using the power grip. Thus, among the four possible grip/goal combinations that were presented, two were biomechanically optimal and two were suboptimal. During the goal-visible movies (bias block) the action lasted until the achievement of its underlying intention (2000 ms). (**b)** The goal-hidden movies (pre-bias and post-bias block) froze 800 ms after the movement onset and the last displayed frame remained on the screen for a duration of 1200 ms so that observers had information about the grip but no further information about the agent’s action intention. Single-pulse TMS was delivered either 600, 700 or 800 ms after movie onset. For each type of movie, participants were required to predict or recognize the intentions that the agent was about to achieve given the type of observed grip. Responses were classified according to whether the intentions they chose were congruent with an OPTIMAL or a SUBOPTIMAL kinematic strategy.

In each movie the handle located on top of the box could either be lifted to open the box or rotated to turn on the light (see Fig. 3a) and the agent could perform each action with either a precision grip or a power grip. These grip/goal combinations were deliberately chosen to ensure that two of them led to biomechanically effortless, optimal kinematics (power grip/open the box; precision grip/turn on the light) while the other two led to biomechanically effortful, suboptimal kinematics (precision grip/open the box; power grip/turn on the light). To verify that our optimal/suboptimal classification reflected the effort that naïve observers associated with each type of action, 10 individuals used a 5-point Likert scale (ranging from 0 = ‘no effort’ to 5 = ‘very big effort’) to rate the biomechanical (muscular and/or articulator) effort required to perform each action^34^. The results of these ratings reflected our *a priori* classification: movies featuring optimal kinematics were estimated as requiring significantly less effort (precision grip/turn-on the light and power grip/open the box, mean score = 1.01) than movies featuring suboptimal kinematics (precision grip/open the box and power grip/turn-on the lights, mean score = 3.13) (two-tailed t-test, t = 220.87, p < 0.0001).

### Manipulating visual uncertainty of action stimuli within the task

A series of 96 *goal-visible* movies (24 movies × 4 grip/action combinations, each movie being unique) was created featuring the agent’s complete action (both the grip and the final goal were apparent, see Fig. 3a) (for more details, see^34^,^55^). From these goal-visible movies we extracted another set of 96 *goal-hidden* movies in which the ‘to-be-achieved’ action goals were made visually uncertain by stopping the video 800 ms after video onset (see Fig. 3b). The last displayed frame remained on the screen for 1200 ms (total duration = 2000 ms) such that the grip (precision or power) but not the agent’s final goal (open or turn) was visible (see Fig. 3b). Goal-hidden movies were displayed twice during the experiment, once before (pre-bias block) and once after (post-bias block) a single block of goal-visible movies (bias block).

### Manipulating probability distributions in the goal-visible movies to bias prior expectations

**Figure 4:**
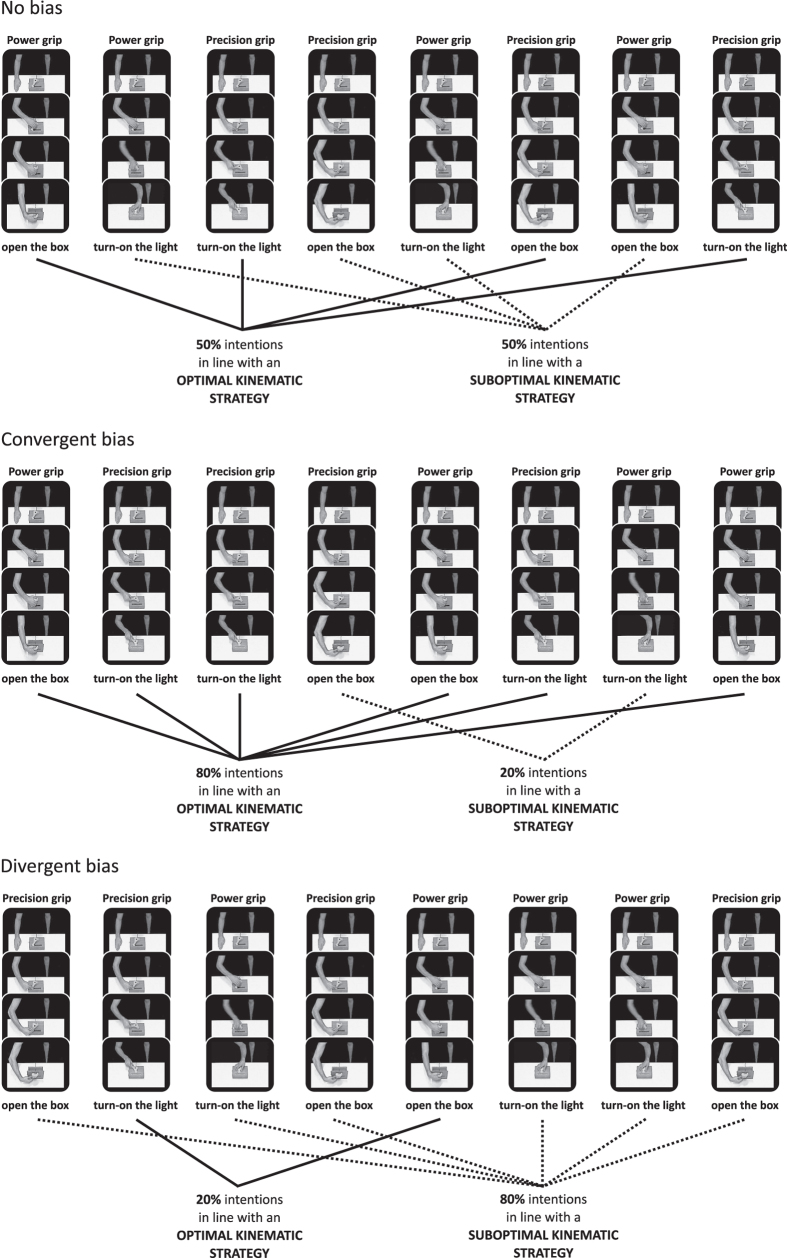
The three types of probabilistic bias. Example of how unbiased and biased probabilities were implemented within a sub-block of eight consecutive goal-visible movies (a full bias block was composed of 12 sub-blocks). Participants were randomly assigned to one of the three bias groups. Participants assigned to the ‘NO BIAS’ group had a 50% probability of observing the agent achieving his intentions using an optimal kinematic strategy (upper panel). Those assigned to the other two groups observed a block of goal-visible movies in which there was an 80% (‘CONVERGENT BIAS’, middle panel) or 20% (‘DIVERGENT BIAS’, lower panel) probability of observing the agent using an optimal kinematic strategy strategy to achieve his goals. Note that in each of these three bias blocks participants had a 50% probability of observing the agent using a power grip, 50% probability of observing a precision grip; 50% probability of observing the agent achieving the ‘open the box’ intention, and 50% probability of observing the ‘turn on the light’ intention.

Participants were randomly assigned to one of three bias groups and unbeknownst to them, each groups differed in terms of the probability of observing the agent achieving goals using an optimal or a suboptimal kinematic strategy. Note that the probability of viewing the ‘open the box’ and the ‘turning on the light’ goals remained equal across groups. Participants assigned to the ‘NO BIAS’ group had a 50% probability of observing the agent achieving his goals using an optimal kinematic strategy. Those assigned to the other groups observed a block of goal-visible movies in which there was an 80% (‘CONVERGENT BIAS’) or 20% (‘DIVERGENT BIAS’) probability of observing the agent using an optimal kinematic strategy to achieve his goals (see Fig. 4). These labels were attributed because the probabilities chosen either *converged* towards or *diverged* away from biomechanical priors.

The 96 goal-visible movies were presented as 12 sub-blocks of 8 videos and the probability distributions for each group (Convergent, Divergent, No Bias) could be extracted from each sub-block. For example, for the Convergent bias group, each sub-block contained 4 precision grip videos (3 precision/turn and 1 precision/open) and 4 power grip videos (3 power/open and 1 power/turn). The order of these 8 videos was randomized separately for each sub-block and all participants in a given group watched the videos in exactly the same order. This block-randomization approach was chosen to avoid any uncontrolled learning biases that might have been unintentionally introduced by a fully randomized design.

Varying the probability distributions of the two types of kinematic strategies allowed us to manipulate the prior expectations each participants formed about the goal the agent was about to achieve given the type of grip he was currently using. At the end of the experiment participants were questioned about the movies, but none of them reported being aware that one type of kinematic strategy was more likely to be observed than another.

### Predicting action goals in the goal-hidden movies before and after biasing prior expectations

**Figure 5:**
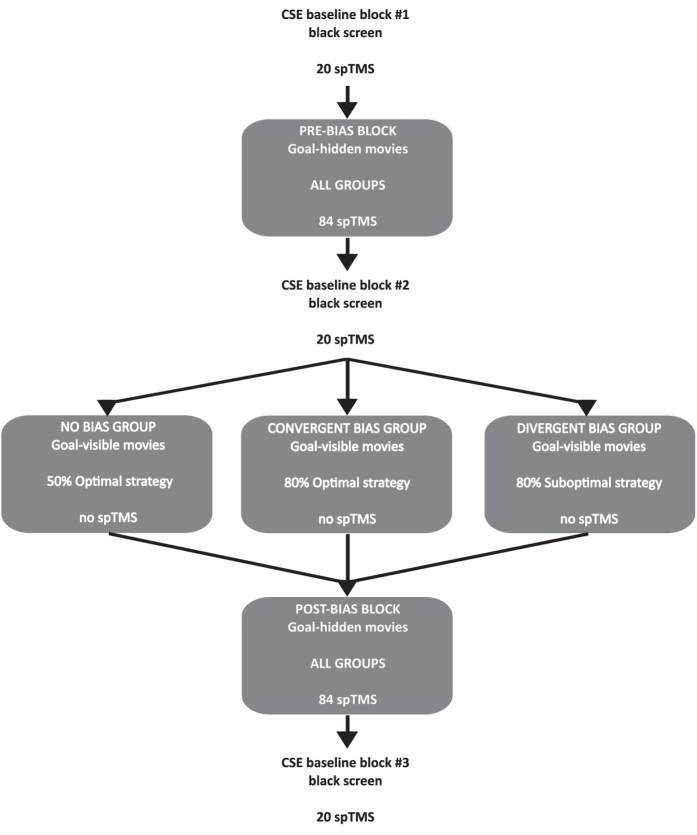
The experimental session. The pre and the post-bias blocks featured goal-hidden movies (high visual uncertainty) where only the grip (not the final goal) remained visible until the end of the trial. TMS was delivered over the left M1 at 600, 700 or 800 ms after the movie onset. Participants were required to predict the final intention of the agent. The bias block featured goal-visible movies (low visual uncertainty) where both the grip and the final intention were visible and participants were required to recognize the final intention of the agent. No TMS was delivered in this block. Across groups, participants were experimentally biased toward one of the two alternative kinematic strategies (convergent bias = 80% optimal kinematics; divergent bias = 20% optimal kinematics) or were not biased at all (no bias = 50% optimal kinematics). The three TMS baseline blocks each consisted of 20 TMS pulses delivered while participants looked at a white cross located at the center of a black screen. MEPs recorded during these blocks were used as a baseline to compute the variation of MEP amplitude during the experiment. Baseline blocks were inserted before and after the pre-bias block, and after the post-bias block.

Participants watched 96 goal-hidden movies before (pre-bias block) and after (post-bias block) their prior expectations were biased by exposure to the goal-visible movies (see Fig. 5). The goal-hidden movies were generated by taking clips from the longer goal-visible movies. Thus, each movie could be identified as belonging to one of the four grip × goal combinations. Participants saw the same number of precision and power grips, and the four grip/goal combinations were presented an equal number of times (even though the final goal was never visible). Both the pre-bias and post-bias blocks were divided into 12 sub-blocks of 8 videos (2 repetitions of each of the 4 grip × (hidden) goal combinations), and the order of these 8 videos was randomized separately for each sub-block. This block randomization was identical for the pre- and post- bias blocks and for all participants.

### TMS and electromyographic (EMG) recording

Motor evoked potentials (MEPs) were recorded from the first dorsal interosseous (FDI) of the right hand, i.e., the mirror of the hand used by the observed agent^85^. FDI was chosen because it is differentially involved in both the precision and power grasping movements used by the agent in the movies^45^. We measured this by asking a naïve participant to watch as many prototypical exemplars of the goal-visible movies as he wanted and then to perform 10 repetitions of each of the four actions (power grip/open the box; power grip/turn on the light; precision grip/open the box; precision grip/turn on the light) while electromyographic activity (EMG) was recorded from his right FDI. On average, there was more activity in FDI during the precision grip than the power grip (0.26 mV (±0.10) vs 0.09 mV (±0.04), t = 5.58, p < 0.001). Another reason for focusing on FDI is that specific changes in FDI’s corticospinal excitability during movement observation have been reported in the literature, whereas the evidence for such changes in other muscles (i.e., abductor digiti minimi muscle – ADM) is meager^86^,^87^,^88^.

EMG recordings were performed using a single differential surface electrode placed over the muscle belly. EMG activity was amplified and digitized using a CED Power 1401 interface (Cambridge Electronic Design, Cambridge, England) and sampled at 5 kHz. Spike2 software (Cambridge Electronic Design, Cambridge, England) was used for off-line data analysis. A Magstim 200^2^ stimulator (The Magstim Company, Carmarthenshire, Wales) generated single-pulse stimuli which were delivered through a figure-of-eight coil (70 mm diameter) placed tangentially to the scalp with the handle pointing backward at a 45° angle away from the midline^89^. Participants wore a tight-fitting bathing cap on which the coil was moved over the left hemisphere to determine the FDI optimal scalp position (OSP). The OSP was then marked on the cap and, together with the coil orientation, was recorded using the SofTaxic Navigator system (EMS, Italy). The coil was hand-held and its position with respect to the target on the standard reconstructed brain was continuously monitored during the experiment.

During the experiment stimulation intensity was set at 120% of the FDI resting motor threshold (rMTs ranged from 29% to 53% of the maximum stimulator output, mean = 40%, SD = 5), defined as the lowest stimulation intensity able to evoke 5 out of 10 MEPs at the OSP with an amplitude of at least 50 μV^90^.

Baseline levels of CSE were established by delivering 20 single TMS pulses while participants viewed a white fixation cross located in the middle of a black screen^25^. Since the experiment lasted nearly 50min, baseline CSE levels were assessed at three different time points: before the pre-bias block, after the pre-bias block (i.e., before the bias block), and after the post-bias block (see Fig. 5).

TMS measurements were made during the pre-bias and the post-bias blocks in which the agent’s goals were hidden (see Fig. 5). The stimulation was applied over the left M1 at either 600, 700, or 800 ms after onset. We chose these timings to ensure that the type of grip had already been fully visible for at least 200 ms and because earlier timings would have decreased our chances of observing muscle-specific CSE modulation^86^,^91^,^92^,^93^,^94^. Moreover, randomly applying TMS at these three times made the TMS delivery as unpredictable as possible given the small time window in which we wanted to simulate. The pre-bias and post-bias blocks contained 12 sub-blocks of 8 movies and TMS was delivered on 7 of the 8 movies in each sub-block. Thus, single-pulse TMS was delivered on approximately 80% of goal-hidden movies (21 MEPs for each of the 4 grip/hidden goal combinations = 84 trials/goal-hidden block), while no pulse was delivered on the remaining 20% (24 trials/goal-hidden block). This procedure allowed us to minimize anticipatory motor activity that could have contaminated the EMG responses evoked by the stimulation.

## Data processing

### Prior expectations

For each movie presented during the experiment participants were required to choose whether the agent was going to open the box by lifting the handle (intention A) or turn-on the light by rotating the handle (intention B). These responses were used to calculate prior expectations^74^,^95^,^96^. The first step in this calculation was to classify the responses as optimal or suboptimal. An ‘optimal’ response refers to the prediction of an intention that is congruent with an optimal (effortless) kinematic strategy, i.e., predicting the ‘open the box’ intention when a power grip is viewed, and predicting the ‘turn on the light’ intention when a precision grip is viewed. A ‘suboptimal’ response refers to the prediction of an intention that is congruent with a suboptimal (effortful) kinematic strategy, i.e., predicting the ‘open the box’ intention when a precision grip is viewed, and predicting the ‘turn on the light’ intention when a power grip is viewed. Before calculating prior expectations and how they changed across the experiment, we first ensured that participants correctly recognized the goals in the goal-visible movies (a prerequisite for integrating the probability bias in these videos). To do this we analyzed the error rates in the goal-visible movies (see Supplementary Materials and Results).

Next, prior expectations were calculated using a simple Bayesian learning scheme (ideal Bayesian observer) in which all marginal and conditional probability estimates were updated after each new event^97^,^98^. Our ideal Bayesian observer was initialized with flat prior distributions at the beginning of the pre-bias and the bias blocks. However, because it is unlikely that participants remember everything they see throughout the task (*ideal observer* model), we modeled their individual limited memory capacity by including a memory decay parameter (*α*) that reduced the weight of past events (*real observer* model). For each new event *e*_*t*_, presented at time step *t*, current values of the marginal probability of the event *i* were defined in the following way:


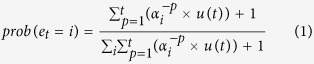


where *p* is the position of a particular action backward from time step *t*, *α*_*i*_^*−p*^ the information weight of action *i* at position *p*, and *u(t)* a binary function indicating whether the optimal (*u*(*t*) = 1) or suboptimal (*u*(*t*) = 0) action intention was chosen at time step *t.* For *α* = 1 (*ideal observer*), there is no information loss and all past actions are weighted equally, while for *α* > 1 (*real observer*), past observations are discounted. The parameter *α* was fit by maximizing the least-square given the data (Optimal Responses, OR) such that


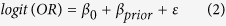


where the standardized parameter estimate *β*_*prior*_ represents the independent contribution of the participant’s prior expectations to the prediction of intentions congruent with optimal kinematics (optimal). The resulting weighted probability estimates were then averaged across all trials of each block and used to obtain a response bias (RB) reflecting the participant’s belief that a certain type of action intention or goal is about to occur given the type of observed grip. Near-zero RB values indicate that a participant is not biased towards either optimal or suboptimal responses, whereas deviations from zero indicate a bias for ‘optimal’ (RB > 0) or ‘suboptimal’ responses (RB < 0). For each participant, the mean of the RB values derived from the *real observer* model was calculated separately for each of the three movie blocks and for each grip. Participants (n = 2) with a RB greater than the mean RB of the entire group (n = 46) by more or less than 2SDs in at least two of these conditions were removed from the analyses. After rejection of these two participants the no bias and convergent bias groups contained 15 participants, while the divergent bias group contained 14 participants.

### Corticospinal excitability (CSE)

CSE was measured as the peak-to-peak amplitude of the MEP in a 50 ms window following the TMS pulse. EMG activity was visually monitored during the experiment to ensure muscle relaxation, and trials were discarded if the root mean squared (RMS) of the EMG during the 200 ms prior to the TMS pulse exceeded the average RMS (of all valid TMS trials in that block) by more than 2SDs. Trials were also excluded if a MEP could not be distinguished from the background EMG or if the peak-to-peak amplitude of the MEP was ±2SDs beyond the mean calculated in each condition of each goal-hidden block. The total percentage of excluded MEPs ranged between 1 and 6% (mean: 3.5%) and was comparable across groups.

MEPs in the first block of goal-hidden movies were expressed as a percentage of the mean MEP amplitude recorded during baseline blocks presented before and after the pre-bias block (baseline 1 = 40 MEPs), while MEPs in the second block of goal-hidden movies were expressed as a percentage of the mean MEP amplitude recorded during baseline blocks presented after the pre-bias block and after the post-bias block (baseline 2 = 40 MEPs) (see Fig. 5). To account for the skewed distribution of MEP ratios all values were then log_10_ transformed^99^ and subsequent analyses were performed on the transformed data.

## Additional Information

**How to cite this article**: Jacquet, P. O. *et al.* Changing ideas about others’ intentions: updating prior expectations tunes activity in the human motor system. *Sci. Rep.* **6**, 26995; doi: 10.1038/srep26995 (2016).

## Supplementary Material

Supplementary Material

## Figures and Tables

**Table 1:** Mean prior expectations (RB) towards optimal (+) or suboptimal (−) action intentions.

	Pre-bias block		Bias block		Post-bias block	
	Grip		Grip		Grip	
	Power	Precision	Power	Precision	Power	Precision
**No bias**	0.12 (±0.02)	0.14 (±0.02)	0.07 (±0.02)	0.05 (±0.01)	0.10 (±0.02)	0.12 (±0.02)
**Convergent bias**	0.16 (±0.02)	0.18 (±0.03)	0.32 (±0.01)	0.32 (±0.01)	0.23 (±0.02)	0.24 (±0.02)
**Divergent bias**	0.12 (±0.03)	0.13 (±0.03)	−0.20 (±0.02)	−0.20 (±0.02)	0.07 (±0.03)	0.05 (±0.03)

Mean prior expectations (±SEM) measured as a response bias (RB) toward intentions congruent with an optimal (positive values) or a suboptimal (negative values) kinematic strategy. Near-zero RB values indicate that on average participants were not biased toward one or the other response type, whereas deviations from zero indicate a bias for ‘optimal’ (RB > 0) or ‘suboptimal’ action intentions (RB < 0).

**Table 2:** Mean log-normalized CSE level for all action prediction conditions.

	Pre-bias block				Post-bias block			
	Grip				Grip			
	Power		Precision		Power		Precision	
	Response				Response			
	Opt	Subopt	Opt	Subopt	Opt	Subopt	Opt	Subopt
**No bias**	2.15 (±0.05)	2.16 (±0.05)	2.15 (±0.05)	2.17 (±0.05)	2.10 (±0.04)	2.09 (±0.04)	2.11 (±0.04)	2.10 (±0.04)
**Convergent bias**	2.14 (±0.06)	2.13 (±0.08)	2.14 (±0.07)	2.12 (±0.08)	2.17 (±0.07)	2.17 (±0.08)	2.15 (±0.07)	2.14 (±0.08)
**Divergent bias**	2.14 (±0.07)	2.13 (±0.07)	2.15 (±0.07)	2.13 (±0.07)	2.00 (±0.08)	2.01 (±0.07)	2.00 (±0.08)	1.98 (±0.07)

Mean log-normalized CSE level (±SEM) for the pre- and post-bias blocks for each of the three groups (no bias, convergent bias, divergent bias groups). Values are presented separately for each observed grip (power, precision) and each response type (Opt = <optimal, Subopt = suboptimal).
