# Supplementary material for: Changing ideas about others’ intentions: updating prior expectations tunes activity in the human motor system
Source: Sci Rep. 2016 May 31;6:26995. doi: 10.1038/srep26995 (PMC4886635; doi:10.1038/srep26995)
Supplement: Supplementary Material [file srep26995-s1.doc]

**Changing ideas about others’ intentions: updating prior expectations tunes activity in the human motor system**

Pierre O. Jacquet^1,2^, Alice C. Roy^3^, Valérian Chambon^2,4^, Anna M. Borghi^5,6^, Roméo Salemme^7,8^, Alessandro Farnè^7,8*^, Karen T. Reilly^7,8*^

^1^ Evolution and Social Cognition Group, Laboratoire de Neurosciences Cognitives (LNC), Département d’Etudes Cognitives, INSERM U960, Ecole Normale Supérieure, PSL Research University, F-75005 Paris, France.

^2^ Evolution and Social Cognition Group, Institut Jean Nicod, Département d’Etudes Cognitives, CNRS UMR8129, Ecole Normale Supérieure, PSL Research University, F-75005 Paris, France.

^3^ Dynamique Du Langage, CNRS UMR 5596, University Lumières Lyon II, 69363 Lyon, France;

^4^ Department of Neuroscience, Biotech Campus-University of Geneva, 1211 Geneva, Switzerland;

^5^ Department of Psychology, Bologna University, 40127 Bologna, Italy;

^6^ Institute of Sciences and Technologies of Cognition, CNR, 00185 Rome, Italy.

^7^ ImpAct Team, Lyon Neuroscience Research Center CRNL, INSERM U1028, CNRS UMR5292, & Hospices Civils de Lyon, Neuro-immersion & Mouvement et Handicap, 69675 Bron, France;

^8^ University Claude Bernard Lyon I, 69100 Villeurbanne, France;

*A.F. and K.T.R. contributed equally to this work

**Supplementary Material**

**Assessing motor facilitation (raw MEPs).** To test whether our goal-hidden movies elicited motor resonance we compared raw MEP amplitudes recorded during the pre-bias block (power grip and precision grip) with those recorded during baseline blocks 1 and 2, using a 4 3 repeated-measures ANOVA with ‘observation condition’ (baseline 1 vs. baseline 2 vs. power grip vs. precision grip) as a within-subject factors and ‘group’ (no bias, convergent bias, divergent bias) as a between-subject factor. This analysis revealed a main effect of the observation condition (F_3,123_ = 17.39, p < .001). First, there was no difference between the mean raw MEP amplitude of the baseline 1 and 2 (1.44 mV ± .9 vs. 1.43 mV ± .9, p > .92), indicating that baseline CSE was stable across groups and across the experiment. Second, mean raw MEP amplitudes when observing the agent’s power (2.12 mV ± 1.37) or precision grip (2.13 mV ± 1.39) (all ps < .001) were greater than in the two baseline blocks, indicating that before being exposed to the probabilistic bias observing the agent grasping using a power or precision grip elicited a motor facilitation effect in all three groups.

**Reaction Times (RTs).** Because a change in Response Bias could also affect the speed with which participants made their predictions we analyzed RTs associated with optimal and suboptimal responses by looking at the factors grip (power and precision) and response type (optimal or suboptimal). Since the motivation for this analysis was to ascertain whether RTs differed according to whether choices were directed toward optimality or suboptimality, both correct predictions and errors were included. This also ensured that RTs for the goal-hidden movies were analysed using the same data sets as those used for the CSE analyses. Post-hoc pairwise comparisons were carried out using Newman-Keuls tests.

RTs recorded during the goal-visible movie block were analysed using a 2 × 2 × 3 repeated-measures ANOVA with ‘grip’ (power, precision) and ‘response type’ (optimal, suboptimal) as within-subject factors and ‘group’ (no bias, convergent bias, divergent bias) as a between-subject factor. This analysis revealed main effects of grip (F_1,41_ = 16.15, p < .001) and response type (F_1,41_ = 65.18, p < .001). Overall, participants responded faster when a precision grip was seen than when a power grip was seen (1385ms vs 1413ms, p < .001). In addition, participants were faster to make optimal responses (‘open the box’ when a power grip was seen / ‘turn-on the light’ when a precision grip was seen) than suboptimal responses (‘open the box’ when a precision grip was seen / ‘turn-on the light’ when a power grip was seen) (1357ms vs. 1441ms, p < .001). There was also an interaction between grip and response type (F_1,41_ = 14.06, p < .001) which was explained by the fact that RTs were slower for suboptimal responses when they viewed a power grip than a precision grip (power grip/turn on light: 1468ms vs. precision grip/open box: 1414ms, p < .001). Note that this grip difference was not observed for optimal responses (1360ms vs. 1356ms, p = .75). More importantly, the analysis revealed a significant interaction between group and response type (F_2,41_ = 24,71, p < .001). Participants exposed to no bias were faster to make optimal responses than suboptimal responses (1369ms vs. 1419ms, p < .05), as were participants exposed to the convergent bias (1318ms vs. 1499ms, p < .001). In contrast, participants exposed to the divergent bias had similar RTs for optimal and suboptimal responses (1387ms vs. 1403ms, p = .38). When compared with participants exposed to no bias, those exposed to the convergent bias tended to make optimal responses faster (1318ms vs 1369ms, p = .08) and suboptimal responses were slower (1419ms vs. 1499ms, p < .01). This difference was not observed when comparing RTs for the no-bias and divergent bias groups (p = .58). Finally, participants in the divergent group tended to be slower than those in the convergent group to make optimal responses (1318ms vs 1387ms, p = .05), and were faster to make suboptimal responses (1499ms vs 1403ms, p < .01).

RTs recorded during the goal-hidden movie blocks were analyzed using a 2 × 2 × 2 × 3 repeated-measures ANOVA with ‘block (pre-bias, post-bias), ‘grip’ (power, precision) and ‘response type’ (optimal, suboptimal) as within-subject factors and ‘group’ (no bias, convergent bias, divergent bias) as a between-subject factor. This analysis showed main effects of grip (F_1,41_ = 17.34, p < .001) and response type (F_1,41_ = 29.65, p < .0001). Participants were faster to predict intentions achieved with a precision grip (1343ms vs 1370ms, p < .001) and to make optimal responses (1335ms vs. 1377ms, p < .001). The interaction between these two factors was also significant (F_1,41_ = 6.10, p < .05). As for the goal-visible movie block, this interaction was explained by the fact that overall, RTs for suboptimal responses were slower when participants viewed a power grip than a precision grip (power/turn on light: 1402ms vs. 1353ms, p < .001). There was no significant interaction between response type and block, suggesting that even though participants changed their pattern of response (reflected by the RB and error rate data) after exposure to a probabilistic bias, their reaction times for optimal and suboptimal responses did not vary.

**Error rates during goal-visible and goal-hidden movie blocks for each possible grip × goal combination.** In order to complement the analysis performed on the participants’ response bias toward biomechanical optimality (RB), we examined the types of errors made by participants by analyzing the error rates associated with each of the four possible grip/visible or hidden goal combinations. The error rate was calculated as the proportion of trials on which the predicted goal differed from the visible or hidden goal. Post-hoc pairwise comparisons were carried out using Newman-Keuls tests.

In a first step we focused on the error rates during the goal-visible movie block and analyzed them using a 2 × 2 × 3 repeated-measures ANOVA with ‘grip’ (power, precision) and ‘visible goal’ (open the box, turn on the light) as within-subject factors and ‘group’ (no bias, convergent bias, divergent bias) as a between-subject factor. The analysis revealed a significant interaction between ‘grip’ and ‘visible goal’ (F_1,41_ = 29.44, p < .0001). Overall, when a power grip was observed, participants made more recognition errors for the ‘turn-on the light’ goal than the ‘open the box’ goal (22% vs. 09%, p = .001). The opposite pattern was obtained with the precision grip, with participants making more errors for the ‘open the box’ goal than the ‘turn on the light’ goal (21% vs. 08%, p < .001). The interaction between grip and goal was further modulated by the group factor (F_2,41_ = 15.40, p < .0001) and post-hoc comparisons showed that while both the no-bias and convergent bias groups exhibited the pattern of errors revealed by the grip × goal interaction described above, this was only significant in the convergent bias group (power grip: 33% vs. 03%, p = .001; precision grip: 32% vs. 03%, p < .001). These differences were not observed in the no bias group (both ps > .26) or the divergent bias group (both ps > .84). Finally, it is important to note that participants in the convergent group tended to make more errors than those in the divergent group when recognizing the ‘turn on the light’ goal achieved with a power grip (33% vs. 13%, p = .06), and the ‘open the box’ goal achieved with a precision grip (32% vs. 14%, p = .05).

In a second step we focused on the error rates during the goal-hidden movie blocks. A 2 × 2 × 2 × 3 repeated-measures ANOVA with ‘block’ (pre-bias, post-bias), ‘grip’ (power, precision) and ‘hidden goal’ (open the box, turn on the light) as within-subject factors and ‘group’ (no bias, convergent bias, divergent bias) as a between-subject factor revealed a significant interaction between our 4 factors (F_2,41_ = 10.16, p < .001). To better understand this interaction, for each block of goal-hidden movies (the first and third columns in Supplementary Fig. S1) we ran a 2 × 2 × 3 repeated-measures ANOVAs with ‘grip’ (power, precision) and ‘hidden goal’ (open the box, turn on the light) as within-subject factors and ‘group’ (no bias, convergent bias, divergent bias) as a between-subject factor. For the pre-bias block, this analysis revealed a significant interaction between ‘grip’ and ‘hidden goal’ (F_1,41_ = 93.46, p < .0001) which was not further modulated by group. When a power grip was observed, participants made more errors for the ‘turn-on the light’ hidden goal than the ‘open the box’ hidden goal (63% vs. 35%, p < .001). The opposite pattern was obtained with the precision grip, with participants making more errors for the ‘open the box’ hidden goal than the ‘turn on the light’ hidden goal (67% vs. 34%, p < .001). This indicates that by-default, participants were biased toward predicting the two goals that were congruent with an optimal kinematic strategy. Importantly, error rates did not differ between these two combinations (power grip/open the box = 64% vs. precision grip/turn on the light = 68%, p = .42), nor between the biomechanically suboptimal combinations (power grip/turn on the light = 35% vs. precision grip/open the box = 34%, p = .74).

The grip × hidden goal interaction was also significant in the post-bias block of goal-hidden movies (F_1,41_ = 92.28, p < .0001), but it was further modulated by the ‘group’ factor (F_2,41_ = 13.14, p < .0001). When a power grip was observed participants in the no bias group tended to make more errors for the ‘turn on the light’ hidden goal than the ‘open the box’ hidden goal (59% vs. 41%, p = .10), and when a precision was observed they made more errors for the ‘open the box’ hidden goal than the ‘turn on the light’ hidden goal (63% vs. 37%, p < .05). This pattern of errors reflects a preference for predicting goals congruent with optimal kinematics, and this preference was even stronger for participants exposed to a convergent bias (power grip: open the box = 26% vs. turn on the light = 77%, p < .001; precision grip: open the box = 73% vs. turn on the light = 21%, p < .001). In contrast, this preference was dampened for participants exposed to a divergent bias as their error rates for the two hidden goals did not differ, either for the power grip (open the box = 39% vs. turn on the light = 58%, p = .09) or the precision grip (open the box = 59% vs. turn on the light = 47%, p = .41). Note that within each of the three groups, error rates for optimal trials were similar for the two trial types (power grip/open the box and precision grip/turn on the light, all ps > .53). This was also the case for suboptimal trials (power grip/turn on the light vs. precision grip/open the box, all ps > .12).

These results show that neither the grip nor the hidden goal affected participants’ predictions. Instead, their predictions were based upon biomechanical optimality, with all three groups behaving similarly before exposure to the probabilistic bias, and each group changing their behavior in accordance with the bias to which they were exposed.

Overall, these results are consistent with those obtained from our response bias analysis. Exposure to biased probabilities during the goal-visible movie block biased participants towards predicting intentions that were achieved using the more frequently observed action strategy. That is, a preference for predicting intentions achieved with an optimal kinematic strategy in the case of a convergent bias and a suboptimal kinematic strategy in the case of a convergent bias. We thus interpret the pattern of incorrect predictions in each group as evidence that participants adaptively up-dated their prior expectations as a result of exposure to the probabilistic bias in the goal-visible movie block.


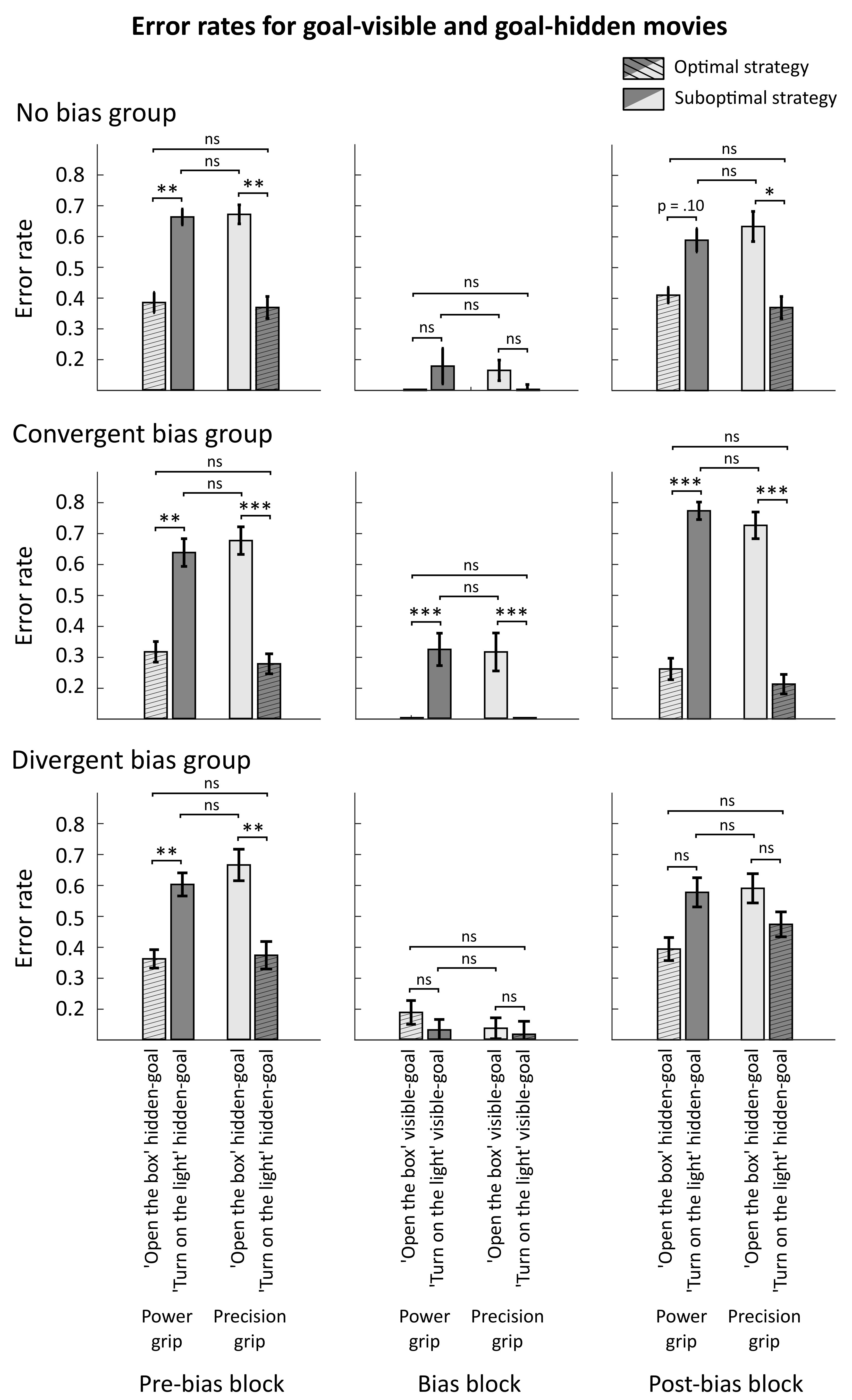


**Supplementary Figure S1. Error rate during the two goal-hidden and the goal-visible movie blocks.** Error rates (y axes) during the goal-hidden movie block presented before (left column), after (right column) the probabilistic exposure, and for the goal-visible movie block (middle column). Errors are presented separately for each type of observed grip and each possible intention (x axes). The hashed columns represent goals (or intentions) that were achieved using an optimal kinematic strategy. For each grip/goal combination, the error rate was calculated as the proportion of trials on which the predicted goal differed from the visible or hidden goal. The Asterisks indicate significant comparisons (* p < .05; ** p < .01; *** p < .001).
